# Does lower extremity pain precede spinal pain? A longitudinal study

**DOI:** 10.1007/s00431-018-3235-6

**Published:** 2018-09-19

**Authors:** Signe Fuglkjær, Werner Vach, Jan Hartvigsen, Niels Wedderkopp, Tina Junge, Lise Hestbæk

**Affiliations:** 1 Department of Sports Science and Clinical Biomechanics, Faculty of Health Sciences, University of Southern Denmark, Campusvej 55, 5230 Odense M, Denmark; 2 Department of Orthopaedics and Traumatoloy, University Hospital Basel, Spitalstr 21, CH-4031 Basel, Switzerland; 3 Institute for Medical Biometry and Statistics, Medical Faculty and Medical Center, University of Freiburg, Freiburg, Germany; 4 Nordic Institute of Chiropractic and Clinical Biomechanics, Campusvej 55, 5230 Odense M, Denmark; 5 Institute of Regional Health Services Research, University of Southern Denmark, Winsloewparken 193, 5000 Odense C, Denmark; 6 Sports Medicine Clinic, Orthopaedic Department, Hospital Lillebaelt, Østre Hougvej 55, 5500 Middelfart, Denmark; 7 Health Sciences Research Centre, University College Lillebaelt, Vestre Engvej 51C, 7100 Vejle, Denmark

**Keywords:** Predictor, Risk factor, Low back pain, Epidemiology, Childhood, Kinetic chain

## Abstract

The main objective was to investigate whether children aged 9–15 years at baseline were more likely to experience an incident event of spinal pain after experiencing lower extremity pain. Children’s musculoskeletal pain was monitored by weekly mobile phone text message responses from parents, indicating whether the child had spinal pain, lower extremity pain, or upper extremity pain the preceding week. Data were analyzed using mixed effect logistic regression models and cox regression models. The association between an incident event of spinal pain and LE pain the preceding weeks increased with increasing observation period and was statistically significant for 12 and 20 weeks (OR = 1.34 (95% CI 1.05 to 1.70) and OR = 1.39 (95% CI 1.11 to 1.75), respectively). We found that the likelihood increased in children with more frequent or longer duration of lower extremity pain. The reversed relationship was investigated as well, and we also found a positive association between spinal pain and a subsequent incidence event of lower extremity pain, but less pronounced.

*Conclusion*: Children were more likely to experience an incident event of spinal pain after experiencing lower extremity pain. The likelihood increased in children with more frequent or longer duration of lower extremity pain.

**Table:** 

**What is Known:** *• Both spinal pain and lower extremity pain often start early in life and is common already in adolescence.*	
**What is New:** *• Children were more likely to experience an incident event of spinal pain after experiencing LE pain.* *• The likelihood increased in children with more frequent or longer duration of LE pain*

**Electronic supplementary material:**

The online version of this article (10.1007/s00431-018-3235-6) contains supplementary material, which is available to authorized users.

## Introduction

Spinal pain often starts early and is common already in adolescence [1, 6, 20], and therefore knowledge about risk factors and predictors should be explored in childhood. Spinal pain has been associated with physical activity [2], overweight [24], widespread pain [18], and most of all, a previous episode of spinal pain [27, 30].

In children, lower extremity (LE) pain is more common than spinal pain [9, 25] with ankle and foot pain being the most common pain sites in young children [12, 16, 31], while knee problems become more prevalent during adolescence [21, 25]. Recently, it has been shown that the prevalence of LE pain decreases from the age of 11 [13], whereas the prevalence of spinal pain increases from about the same age [6, 20]. We therefore hypothesize that LE pain in young children may predispose the child for subsequent spinal pain. This might be due to pain-induced changes in movement patterns, which could lead to altered biomechanical loading in other regions and thereby cause compensatory pain. Thus potentially, a change in the kinetic chain between the LEs and the spine could lead to spinal pain, and indeed indications of an association between LE pain and later spinal pain have been found previously [23, 26].

However, the presence of co-occurring musculoskeletal pain is fairly common, also in children [8, 14, 15, 25], and it is well known that pain in one site is a strong predictor for pain elsewhere. Several theories have been explored to explain this phenomenon. A relationship between physical factors, such as overweight or physical activity, and development of spinal pain has high face validity, but the exact nature of such associations remains unclear [19]. Other possible explanations have been proposed, and some examples are (1) central sensitization caused by long-term pain in which altered signaling in the central nervous system amplifies the overall pain perception [22]; (2) several psychological factors have been shown to predict musculoskeletal pain in children [5, 7, 28]; and (3) hypermobility has been proposed as an explanation for spreading of pain sites in girls [8]. However, common for these potential explanations is that they would be expected to affect a potential relationship between pain sites equally in both directions.

To test the hypothesis that LE may increase the risk of subsequent spinal pain, we investigated whether children were more likely to experience an incident event of spinal pain after experiencing LE pain in the preceding weeks and to which degree a potential association depended on the frequency of LE pain prior to the spinal pain. Furthermore, to test whether a potential association was bidirectional, we also estimated the reverse relationship: whether spinal pain would lead to subsequent LE pain and to which degree a potential association depended on the frequency of prior spinal pain.

## Materials and methods

### Setting

This was a prospective school-based cohort study nested within the Childhood Health, Activity and Motor Performance School Study (CHAMPS study-DK). The CHAMPS study-DK was a dynamic cohort study; thus, children could enter and leave the study at any time during the study period. The main purpose of the CHAMPS study-DK was to evaluate the effect of extra physical education on general childhood health. Schools were divided into two groups: intervention schools received six lessons of physical education per week, whereas control schools received two lessons per week. The CHAMPS study-DK is described in detail elsewhere [32]. In this paper, only information with regard to spinal and lower extremity pain will be analyzed.

### Study population

There is evidence that the frequency of spinal pain increase with age [4, 6, 10, 20]. To obtain a satisfying frequency of spinal pain, only data from the last 2 years of the study period (from August 2012 to June 2014) was used in this study. In August 2012, the included pupils attended fourth to eighth grades in 13 out of 17 public primary schools in the municipality of Svendborg, Denmark. This municipality has 58,000 inhabitants and is comparable to the rest of Denmark in terms of age, sex, and income, but has a slightly higher unemployment rate (5.3% versus 4.5%) [29]. In Svendborg, 84% of the children attend public schools, which therefore represent all socioeconomic levels.

### Data collection

Registration of MSK pain was conducted by weekly mobile phone text message responses (SMS responses) from parents. Every week, parents received the following mobile phone text message question (SMS question): “Has [name of the child] had any pain during the past week in: 1-Neck or back; 2-Shoulder, arm or hand; 3-Hip, leg or foot; 4-No, [name of the child] did not have any pain.” It was possible to report more than one pain area. If parents did not reply, they received reminders twice with an interval of 48 h. The SMS question was sent out every week except for 6 weeks during the summer holidays (July and August) and 1 week during the Christmas holidays. If parents texted a “1,” “2,” and/or “3” for the MSK pain question, they were telephoned within 5 days by a member of the clinical team, consisting of licensed and experienced chiropractors and physiotherapists. A standardized interview was performed about the nature of their child’s pain, including information about location and duration of pain and mode of onset.

### Variables of interest

Explanatory variables were LE pain the preceding weeks defined as at least one episode with LE pain within the preceding 1, 2, 4, 8, 12, or 20 weeks, and spinal pain the preceding weeks defined as at least one episode with spinal pain within the preceding 1, 2, 4, 8, 12, or 20 weeks.

Outcome variables were an incident event of spinal pain defined as spinal pain after at least 20 weeks without spinal pain, and an incident event of LE pain defined as LE pain after at least 20 weeks without LE pain. Confounders were age (August 2012) and sex.

### Statistical analyses

Children were included in the final analyses, if SMS responses were available in at least 85% of the weeks from August 2012 to June 2014, excluding the summer holiday of 2013. Potential differences in demographics were tested both between participants and non-participants (children that either refused to participate or never answered the invitation), and between the study sample and children excluded due to low response rate. For non-participants, sex could be determined from the names, but no other information was available.

Analyses were performed at the level of the single weeks. The outcome measurement used in the primary analysis was an incident event of spinal pain, defined as spinal pain after at least 20 weeks without spinal pain. Data included in the analyses were collected from August 2012 until June 2014, and included outcome measurements from January 2013 to June 2013, and from January 2014 to June 2014, to ensure that history of spinal pain of the last 20 weeks in each child was not disrupted by the summer holiday period. Thus, from January 2013 to June 2013 and from January 2014 to June 2014, we considered the probability to experience an incident event of spinal pain in dependence of LE pain within the preceding 1, 2, 4, 8, 12, or 20 weeks. The association between this exposure and the occurrence of an incident event of spinal pain was described by odds ratios (ORs) with 95% confidence interval (CI), obtained from a mixed effect logistic regression with child as a random effect, adjusted for age and sex.

Next, we explored the proportion of weeks with LE pain as a risk factor for incident events of spinal pain using two approaches. First, information about the proportion of weeks with LE pain during the preceding 20 weeks was calculated, and for each week, children were categorized into three groups: No LE pain within the preceding 20 weeks, LE pain 1–50% of the weeks, LE pain > 50% of the weeks. Again, associations were expressed by OR with 95% CI obtained from a mixed effect logistic regression model with child as a random effect. Trends were assessed by considering the three categories as a continuous covariate coded as 1, 2, and 3.

We then performed a time-to-event analysis to investigate whether the time to experience an incident event of spinal pain (event of interest) depended on the proportion of weeks with LE pain prior to the beginning of analysis. We considered two starting times: January 1, 2013, and January 1, 2014, and combined the analyses. Children with spinal pain within 20 weeks prior to each start date were excluded from the analysis. Children were categorized into three groups based on proportion of weeks with LE pain 20 weeks prior to the start date: No LE pain within the preceding 20 weeks LE pain 1–50% of the weeks, LE pain > 50% of the weeks. Kaplan–Meier curves were used to visualize the time until an incident event of spinal pain. Differences between the curves were expressed as Hazard ratios with 95% CIs based on a Cox model, adjusted for sex and age. Again, test for trend was included. Clustering due to using children potentially experiencing more than one event was taken into account by using robust standard errors.

All analyses were repeated stratified by sex.

Missing SMS responses were imputed as “no pain.” To estimate the impact of this decision, we performed a sensitivity analysis. Missing SMS responses were imputed as the same value as the previous week’s SMS response.

Finally, all analyses were repeated in an identical way, but in the reversed direction. Here, we explored whether the probability to experience an incident event of LE pain, depended on the presence of spinal pain or the proportion of weeks with spinal pain the preceding weeks. Difference in effect estimates between the primary and reversed analyses was assessed by fitting the two models simultaneously and using robust standard errors taking the clustering within children into account.

STATA 15.0 (StataCorp, College Station, TX, USA) was used for analyses, significance level was set at 0.05.

#### Availability of data and materials

Data are available only upon request from the CHAMPS study-DK Steering Committee due to legal and ethical restrictions. Interested parties may contact Dr. Niels Wedderkopp (nwedderkopp@health.sdu.dk) and the following information will be required at the time of application: a description of how the data will be used, securely managed, and permanently deleted.

## Results

### Study sample

From 2011 to 2014, 1917 children were invited to the CHAMPS study-DK and 1465 (76%) accepted participation. From August 2012 to June 2014, 1346 children were included in the study; however, 326 were subsequently excluded due to low SMS compliance, which left 1020 children for the final analyses. There was no significant difference in relation to sex, neither between participants and non-participants nor between compliers and non-compliers. Non-compliers were slightly older than compliers (11.7 years of age versus 11.5 years of age, *p* = 0.03).

Children were 9–15 years (mean age 11.5 years). The majority were girls (Table 1).

**Table 1:** Overview of participants from a cohort of Danish school children; CHAMPS study-DK

Age, August 2012	Boys, *n* (%)	Girls, *n* (%)	Total *n* (%)
	490 (48.0)	530 (52.0)	1020 (100.0)
9	29 (41.4)	41 (58.6)	70 (6.9)
10	90 (41.5)	127 (58.5)	217 (21.3)
11	114 (49.1)	118 (50.9)	232 (22.8)
12	120 (55.0)	98 (45.0)	218 (21.4)
13	86 (46.2)	100 (53.8)	186 (18.2)
14–15	51 (52.6)	46 (47.4)	97 (9.5)
Mean age (SD)	11.6 (SD 1.4)	11.4 (SD 1.4)	11.5 (SD 1.4)

In total, parents of the 1020 children delivered 99,856 SMS responses from August 2012 to June 2014. The majority of the SMS responses was “no pain” (76.0%), 9.2% was missing, of which 6.1% represented the summer holiday. The most commonly reported pain site was ‘LE pain,” which was reported 9548 times (9.6%).

### Is LE pain within the preceding weeks associated with an incident event of spinal pain?

In the primary analysis, the total number of incident events of spinal pain was 301. The association between an incident event of spinal pain and LE pain the preceding weeks increased with increasing observation period and was statistically significant for 12 and 20 weeks (OR = 1.34 (95% CI 1.05 to 1.70) and OR = 1.39 (95% CI 1.11 to 1.75), respectively) (Table 2).

**Table 2:** Age- and sex-adjusted associations, including confidence intervals (CI), between incident events of spinal pain and lower extremity pain the preceding weeks, and the reversed association; from a cohort of Danish school children. Reference is children without lower extremity^1^ or spinal pain^2^

Primary analyses: outcome spinal pain^1^		Reversed analyses: outcome lower extremity pain^2^		*p* value for the difference between primary and reversed analyses
Lower extremity pain within the preceding	Odds ratio (95% CI)	Spinal pain within the preceding:	Odds ratio (95% CI)	
1 week	0.87 (0.58 to 1.31)	1 week	1.26 (0.84 to 1.87)	0.19
2 weeks	0.93 (0.65 to 1.33)	2 weeks	1.14 (0.79 to 1.65)	0.41
4 weeks	1.11 (0.82 to 1.49)	4 weeks	1.18 (0.86 to 1.62)	0.75
8 weeks	1.23 (0.94 to 1.59)	8 weeks	1.09 (0.82 to 1.44)	0.50
12 weeks	1.34 (1.05 to 1.70)	12 weeks	1.15 (0.89 to 1.47)	0.34
20 weeks	1.39 (1.11 to 1.75)	20 weeks	1.12 (0.89 to 1.40)	0.14

^1^Is lower extremity pain within the preceding weeks associated with an incident event of spinal pain?

^2^Is spinal pain within the preceding weeks associated with an incident event of lower extremity pain?

Using information about the proportion of weeks with LE pain during the preceding 20 weeks, we found that children with more frequent or longer duration of LE pain were more likely to experience an incident event of spinal pain than children with less LE pain (Table 3).

**Table 3:** Age- and sex-adjusted associations, including confidence intervals (CI), between incident events of spinal pain and proportion of weeks with lower extremity pain within the preceding 20 weeks, and the reversed association; from a cohort of Danish school children

Primary analyses: outcome spinal pain^1^		Reversed analyses: outcome lower extremity pain^2^		*p* value for the difference between primary and reversed analyses
Proportion of weeks with lower extremity pain	Odds ratio (95% CI)	Proportion of weeks with spinal pain	Odds ratio (95% CI)	
0%	1.00	0%	1.00	
1–50%	1.35 (1.06 to 1.72)	1–50%	1.15 (0.90 to 1.45)	0.29
51–100%	1.59 (1.04 to 2.43)	51–100%	0.94 (0.51 to 1.72)	0.11
Trend	1.30 (1.10 to 1.54)		1.07 (0.91–1.26)	0.09

^1^Is lower extremity pain within the preceding weeks associated with an incident event of spinal pain?

^2^Is spinal pain within the preceding weeks associated with an incident event of lower extremity pain?

**Fig. 1:**
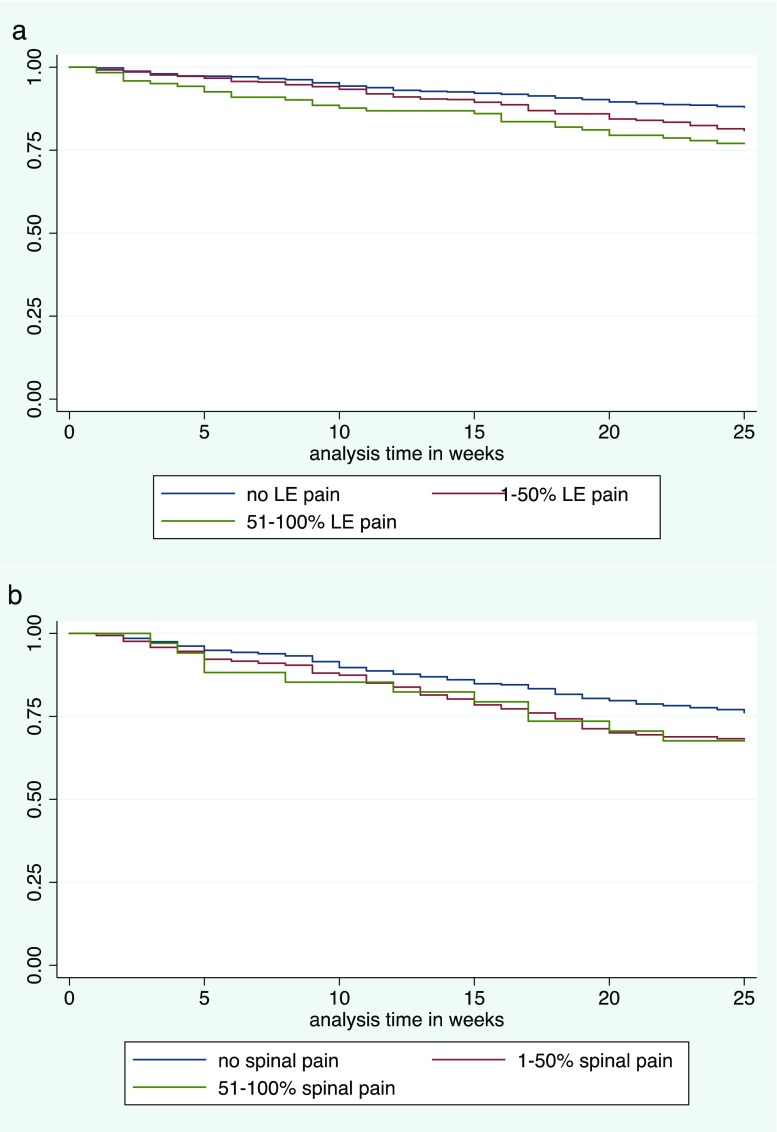
Kaplan–Meier plot illustrating the **a** probability to experience an incident event of spinal pain in dependence of proportion of weeks with lower extremity pain 20 weeks prior to the start of analysis and **b** probability to experience an incident event of LE pain in dependence of proportion of weeks with spinal pain 20 weeks prior to the start of analysis. LE lower extremity

In the time-to-event analysis, 198 and 197 children were excluded due to report of spinal pain from August 2012 to December 2012 and from August 2013 to December 2013, respectively. In total, 822 children for 2013 and 823 for 2014 were used in the time-to-event analysis representing 251 incident events of spinal pain in 234 children. The Kaplan–Meyer plot illustrated that the probability to experience an incident event of spinal pain was higher for the children with LE pain than in the children without LE pain (Fig. 1a).

In the Cox regression analyses, children with LE pain were 1.70 times (95% CI 1.33 to 2.19) more likely to experience an incident event of spinal pain than children without LE pain. Children with a more frequent or longer duration of LE pain were more likely to experience an incident event of spinal pain than children without LE pain (Table 4). Thus, also the time-to-event analysis indicated that the likelihood increased in children with more frequent or longer duration of LE pain.

**Table 4:** Age- and sex-adjusted associations, including confidence intervals (CI), between incident events of spinal pain and proportion of reported lower extremity pain 20 weeks prior to analyses, and the reversed association. From a cohort of Danish school children

Primary analysis: outcome spinal pain ^1^			Reversed analysis: outcome lower extremity pain^2^			*p* value for the difference between primary and reversed analyses
Proportion of weeks with lower extremity pain^1^	*n*	Hazard ratio (95% CI)	Proportion of weeks with lower extremity pain^2^	*n*	Hazard ratio (95% CI)	
0%	1008	1.00	0%	1006	1.00	
1–50%	515	1.62 (1.24 to 2.11)	1–50%	168	1.42 (1.06 to 1.92)	0.51
51–100%	122	2.07 (1.34 to 3.20)	51–100%	34	1.51 (0.82 to 1.78)	0.40
Trend	1645	1.48 (1.24 to 1.89)		1208	1.32 (1.06 to 1.65)	0.38

^1^Is lower extremity pain within the preceding weeks associated with an incident event of spinal pain?

^2^Is spinal pain within the preceding weeks associated with an incident event of lower extremity pain?

### Is spinal pain within the preceding weeks associated with an incident event of LE pain?

In the reversed analysis, the total number of incident events of LE pain was 458. We found a positive, but not statistically significant, association between spinal pain the preceding 20 weeks and the likelihood to experience an incident event of LE pain (Table 2). The number of weeks with spinal pain during the preceding 20 weeks did not seem to influence the risk of an incident event of LE pain (Table 3).

The time-to-event analysis included 590 children for 2013 and 618 children for 2014, representing 308 incident events of LE pain in 282 children. The Kaplan–Meyer plot illustrated that children with spinal pain experienced more incident events of LE pain than children without spinal pain (Fig. 1b).

Children with spinal pain were 1.44 (95% CI 1.09 to 1.90) times more likely to experience an incident event of LE pain than children without spinal pain; however, we did not find that children with more frequent or longer duration of spinal pain were more likely to experience an incident event of LE pain (Table 4).

None of the differences between the primary and the reversed analyses were significant.

### Sex differences

When the primary analyses were stratified for sex, the patterns and associations were accentuated for the girls. In the stratified reversed analyses, there was no evidence of associations for the girls, and some associations for the boys, but without trends in relation to time or frequency of spinal pain. However, it should be noted that the cell sizes for children with pain in more than half of the weeks were small, and the estimates therefore potentially imprecise (Table 5).

**Table 5:** Associations stratified by sex and adjusted for age, including confidence intervals (CI), between incident events of spinal pain and lower extremity pain the preceding weeks, and the reversed association; from a cohort of Danish school children (530 girls and 490 boys)

Primary analyses: outcome spinal pain^1^				Reversed analyses: outcome lower extremity pain^2^					
Lower extremity pain within the preceding:	Girls		Boys		Spinal pain within the preceding:	Girls		Boys	
	*n*	Odds ratio (95% CI)	*n*	Odds ratio (95% CI)		*n*	Odds ratio (95% CI)	*n*	Odds ratio
1 week	291	0.80 (0.45 to 1.41)	276	0.96 (0.53 to 1.73)	1 week	198	1.11 (0.66 to 1.88)	166	1.50 (0.81 to 2.76)
2 weeks	299	0.89 (0.54 to 1.46)	281	0.97 (0.58 to 1.62)	2 weeks	201	0.98 (0.59 to 1.61)	169	1.41 (0.82 to 2.44)
4 weeks	310	1.13 (0.75 to 1.72)	293	1.07 (0.69 to 1.66)	4 weeks	209	1.01 (0.66 to 1.57)	178	1.43 (0.90 to 2.27)
8 weeks	325	1.32 (0.92 to 1.89)	314	1.14 (0.78 to 1.65)	8 weeks	229	1.02 (0.70 to 1.49)	189	1.18 (0.78 to 1.79)
12 weeks	336	1.46 (1.04 to 2.04)	325	1.23 (0.87 to 1.73)	12 weeks	242	0.98 (0.69 to 1.40)	199	1.37 (0.95 to 1.95)
20 weeks	360	1.58 (1.14 to 2.18)	353	1.23 (0.90 to 1.70)	20 weeks	262	0.92 (0.67 to 1.27)	218	1.38 (1.00 to 1.90)
Proportion of weeks with lower extremity pain					Proportion of weeks with spinal pain				
0%	486	1.00	457	1.00	0%	507	1.00	482	1.00
1–50%	347	1.57 (1.11 to 2.22)	347	1.18 (0.84 to 1.66)	1–50%	257	0.91 (0.64 to 1.29)	214	1.45 (1.05 to 2.00)
> 50%	104	1.61 (0.92 to 2.81)	71	1.61 (0.84 to 1.65)	> 50%	58	0.98 (0.50 to 1.92)	21	0.67 (0.17 to 2.72)
Hazard ratio (95% CI)					Hazard ratio (95% CI)				
0%	551	1.00	328	1.00	0%	349	1.00	328	1.00
1–50%	195	1.88 (1.27 to 2.80)	221	1.42 (0.99 to 2.04)	1–50%	84	1.33 (0.85 to 2.05)	70	1.53 (1.02 to 2.31)
> 50%	63	2.54 (1.27 to 2.80)	39	1.54 (0.77 to 3.09)	> 50%	21	2.10 (1.08 to 4.09)	8	0.38 (0.05 to 2.73)

^1^Is lower extremity pain within the preceding weeks associated with an incident event of spinal pain?

^2^Is spinal pain within the preceding weeks associated with an incident event of lower extremity pain?

### Sensitivity analysis

Similar results were found, when missing SMS responses were imputed as the same value as the previous week’s SMS response (Supplementary File 1).

## Discussion

We found that children were more likely to experience an incident event of spinal pain after experiencing LE pain 12 or 20 weeks prior to the spinal pain and this likelihood increased in children with more frequent or longer duration of LE pain. In the reverse analysis, we also found a positive association between spinal pain and the likelihood to experience an incident event of LE pain, but this was less pronounced and there were no patterns in relation to timing or amount of prior spinal pain. The association was more pronounced in girls than in boys. Several factors could explain this finding, e.g., higher muscle strength and endurance in boys than girls [11], or sex-related differences in types of physical activity or in psychological reactions to pain. However, it could be a chance finding since cell sizes are small in the reversed analysis. This is supported by the fact that there was no evidence of association in the reversed analysis, which does not correlate with existing literature showing that more girls than boys experience multisite pain [14, 25].

The primary analysis showed that the risk of spinal pain increased with the amount of LE pain preceding spinal pain. Likewise, significant associations with spinal pain were only seen after more than eight weeks indicating a certain latency period. Since this was not the case in the reversed analysis, this may support the hypothesis of alterations of loading in the development of spinal pain, e.g., certain duration of LE pain may lead to compensatory pain in the spine, possibly due to changes in the kinetic chain. Recently, a similar pattern (increased risk of low back pain in case of LE pain) was found in American soldiers [26].

These findings highlight the importance of a clinical history and examination of the complete MSK system, i.e., children with spinal pain should also receive a thorough examination of the LEs and vice versa.

Mobile phone text messages are known to be a practical and user-friendly method of data collection [3, 17]. In this study, few responded with pain in more than one region. It is unknown if parents were reluctant to report more than one pain site, in which case, the SMS responses may not illustrate the complete picture of MSK pain. Likewise, parents may have been reluctant to report a shift in region of pain and therefore observe the child for a week or two before reporting a new pain episode, and this could add to the lack of association when considering LE pain in the past few weeks.

Major strengths include the large prospective population-based cohort, the high response rate and the short recall period. This study confirms that some individuals are more prone to experience MSK pain than others, and that pain might spread to other areas of the body, but not in a completely haphazard way. Furthermore, these pain patterns tend to start early in life, and thus highlights the importance of development of prevention strategies and effective treatment for MSK pain early in life.

## Conclusion

Children were more likely to experience an incident event of spinal pain after experiencing LE pain. The likelihood increased in children with more frequent or longer duration of LE pain, and was more pronounced in girls than in boys. In the reversed analyses, we also found a positive association between spinal pain and incidence events of lower extremity pain, but this was less pronounced. This paper confirms that attention to the entire musculoskeletal system is required to understand the course and development of spinal pain.

## Electronic supplementary material

ESM 1 (DOCX 106 kb)

## Abbreviations

CHAMPS study-DK: Childhood Health, Activity and Motor Performance School Study
LE: Lower extremity
MSK: Musculoskeletal
SMS: Mobile phone text message
UE: Upper extremity

## References

[1] Aartun E, Hartvigsen J, Wedderkopp N, Hestbaek L (2014). Spinal pain in adolescents: prevalence, incidence, and course: a school-based two-year prospective cohort study in 1,300 Danes aged 11-13. BMC Musculoskelet Disord.

[2] Aartun E, Boyle E, Hartvigsen J, Ferreira PH, Maher CG, Ferreira ML, Hestbaek L (2016). The most physically active Danish adolescents are at increased risk for developing spinal pain: a two-year prospective cohort study. BMJ Open Sport Exerc Med.

[3] Axen I, Bodin L, Bergstrom G, Halasz L, Lange F, Lovgren PW, Rosenbaum A, Leboeuf-Yde C, Jensen I (2012). The use of weekly text messaging over 6 months was a feasible method for monitoring the clinical course of low back pain in patients seeking chiropractic care. J Clin Epidemiol.

[4] Calvo-Munoz I, Gomez-Conesa A, Sanchez-Meca J (2013). Prevalence of low back pain in children and adolescents: a meta-analysis. BMC Pediatr.

[5] Diepenmaat AC, van der Wal MF, de Vet HC, Hirasing RA (2006). Neck/shoulder, low back, and arm pain in relation to computer use, physical activity, stress, and depression among Dutch adolescents. Pediatrics.

[6] Dissing KB, Hestbaek L, Hartvigsen J, Williams C, Kamper S, Boyle E, Wedderkopp N (2017). Spinal pain in Danish school children - how often and how long? The CHAMPS study-DK. BMC Musculoskelet Disord.

[7] Dunn KM, Jordan KP, Mancl L, Drangsholt MT, Le Resche L (2011). Trajectories of pain in adolescents: a prospective cohort study. Pain.

[8] El-Metwally A, Salminen JJ, Auvinen A, Kautiainen H, Mikkelsson M (2004). Prognosis of non-specific musculoskeletal pain in preadolescents: a prospective 4-year follow-up study till adolescence. Pain.

[9] El-Metwally A, Salminen JJ, Auvinen A, Macfarlane G, Mikkelsson M (2007). Risk factors for development of non-specific musculoskeletal pain in preteens and early adolescents: a prospective 1-year follow-up study. BMC Musculoskelet Disord.

[10] Franz C, Wedderkopp N, Jespersen E, Rexen CT, Leboeuf-Yde C (2014). Back pain in children surveyed with weekly text messages - a 2.5 year prospective school cohort study. Chiropr Man Therap.

[11] Fredriksen PM, Mamen A, Hjelle OP, Lindberg M (2018). Handgrip strength in 6-12-year-old children: the health oriented pedagogical project (HOPP). Scand J Public Health.

[12] Fuglkjaer S, Dissing KB, Hestbaek L (2017). Prevalence and incidence of musculoskeletal extremity complaints in children and adolescents. A systematic review. BMC Musculoskelet Disord.

[13] Fuglkjaer S, Hartvigsen J, Wedderkopp N, Boyle E, Jespersen E, Junge T, Larsen LR, Hestbaek L (2017). Musculoskeletal extremity pain in Danish school children - how often and for how long? The CHAMPS study-DK. BMC Musculoskelet Disord.

[14] Hoftun GB, Romundstad PR, Zwart JA, Rygg M (2011). Chronic idiopathic pain in adolescence--high prevalence and disability: the young HUNT study 2008. Pain.

[15] Holden S., Rathleff M.S., Roos E.M., Jensen M.B., Pourbordbari N., Graven-Nielsen T. (2017). Pain patterns during adolescence can be grouped into four pain classes with distinct profiles: A study on a population based cohort of 2953 adolescents. European Journal of Pain.

[16] Jespersen E, Rexen CT, Franz C, Moller NC, Froberg K, Wedderkopp N (2015). Musculoskeletal extremity injuries in a cohort of schoolchildren aged 6-12: a 2.5-year prospective study. Scand J Med Sci Sports.

[17] Johansen B, Wedderkopp N (2010). Comparison between data obtained through real-time data capture by SMS and a retrospective telephone interview. Chiropr Osteopat.

[18] Jones GT, Macfarlane GJ (2009). Predicting persistent low back pain in schoolchildren: a prospective cohort study. Arthritis Rheum.

[19] Kamper SJ, Yamato TP, Williams CM (2016). The prevalence, risk factors, prognosis and treatment for back pain in children and adolescents: an overview of systematic reviews. Best Pract Res Clin Rheumatol.

[20] Kjaer P, Wedderkopp N, Korsholm L, Leboeuf-Yde C (2011). Prevalence and tracking of back pain from childhood to adolescence. BMC Musculoskelet Disord.

[21] Michaleff ZA, Campbell P, Protheroe J, Rajani A, Dunn KM (2017). Consultation patterns of children and adolescents with knee pain in UK general practice: analysis of medical records. BMC Musculoskelet Disord.

[22] O’Neill S, Manniche C, Graven-Nielsen T, Arendt-Nielsen L (2014). Association between a composite score of pain sensitivity and clinical parameters in low-back pain. Clin J Pain.

[23] O'Leary CB, Cahill CR, Robinson AW, Barnes MJ, Hong J (2013). A systematic review: the effects of podiatrical deviations on nonspecific chronic low back pain. J Back Musculoskelet Rehabil.

[24] Paulis WD, Silva S, Koes BW, van Middelkoop M (2014). Overweight and obesity are associated with musculoskeletal complaints as early as childhood: a systematic review. Obes Rev.

[25] Rathleff MS, Roos EM, Olesen JL, Rasmussen S (2013). High prevalence of daily and multi-site pain--a cross-sectional population-based study among 3000 Danish adolescents. BMC Pediatr.

[26] SEAY JOSEPH F., SHING TRACIE, WILBURN KRISTEN, WESTRICK RICHARD, KARDOUNI JOSEPH R. (2018). Lower-Extremity Injury Increases Risk of First-Time Low Back Pain in the US Army. Medicine & Science in Sports & Exercise.

[27] Stahl M, Kautiainen H, El-Metwally A, Hakkinen A, Ylinen J, Salminen JJ, Mikkelsson M (2008). Non-specific neck pain in schoolchildren: prognosis and risk factors for occurrence and persistence. A 4-year follow-up study. Pain.

[28] Stallknecht SE, Strandberg-Larsen K, Hestbaek L, Andersen AN (2017). Spinal pain and co-occurrence with stress and general well-being among young adolescents: a study within the Danish National Birth Cohort. Eur J Pediatr.

[29] Statistics Denmark. http://www.statistikbanken.dk

[30] Taylor JB, Goode AP, George SZ, Cook CE (2014). Incidence and risk factors for first-time incident low back pain: a systematic review and meta-analysis. Spine J.

[31] Verhagen E, Collard D, Paw MC, van Mechelen W (2009). A prospective cohort study on physical activity and sports-related injuries in 10-12-year-old children. Br J Sports Med.

[32] Wedderkopp N, Jespersen E, Franz C, Klakk H, Heidemann M, Christiansen C, Moller NC, Leboeuf-Yde C (2012). Study protocol. The childhood health, activity, and motor performance school study Denmark (the CHAMPS-study DK). BMC Pediatr.

