# Supplementary material for: Does lower extremity pain precede spinal pain? A longitudinal study
Source: Eur J Pediatr. 2018 Sep 19;177(12):1803–10. doi: 10.1007/s00431-018-3235-6 (PMC6244689; doi:10.1007/s00431-018-3235-6)
Supplement: Supplementary file 1 — (DOCX 106 kb) [file 431_2018_3235_MOESM1_ESM.docx]

**Supplementary file 1**

**Imputation of missing mobile phone text message responses (SMS responses)**

A sensitivity analysis was performed to estimate the impact of the decision of imputing missing SMS responses as ‘no pain’, by comparing two different approaches

1. ‘Missing imputed as ‘no pain’ (used in the analyses).

2. ‘Missing imputed as the same SMS response as last week, thus potentially pain.

| Sensitivity analyses: imputation of missing data using two approaches.  Age and sex adjusted associations, including confidence intervals (CI), between incident events of spinal pain and lower extremity (LE) pain the preceding weeks, and the reversed association. | | | | |  |
| --- | --- | --- | --- | --- | --- |
|  | Missing imputed as  ‘no pain’ | | Missing imputed as the same as last week | | |
|  | Odds ratio | (95% CI) | Odds ratio | (95% CI) | |
| **Primary Analysis^1^** |  |  |  |  | |
| LE pain the preceding week | 0.87 | (0.58-1.31) | 0.54 | (0.32-0.91) | |
| LE pain within the preceding 2 weeks | 0.93 | (0.65-1.33) | 0.63 | (0.41-0.97) | |
| LE pain within the preceding 4 weeks | 1.11 | (0.82-1.49) | 0.84 | (0.59-1.18) | |
| LE pain within the preceding 8 weeks | 1.23 | (0.94-1.59) | 1.06 | (0.80-1.39) | |
| LE pain within the preceding 12 weeks | 1.34 | (1.05-1.70) | 1.18 | (0.92-1.52) | |
| LE pain within the preceding 20 weeks | 1.39 | (1.11-1.75) | 1.32 | (1.05-1.67) | |
| **Reversed analyses^2^** |  |  |  |  | |
| Spinal pain within the preceding week | 1.26 | (0.84-1.87) | 1.02 | (0.64-1.62) | |
| Spinal pain within the preceding 2 weeks | 1.14 | (0.79-1.65) | 1.10 | (0.74-1.63) | |
| Spinal pain within the preceding 4 weeks | 1.18 | (0.86-1.62) | 1.15 | (0.82-1.61) | |
| Spinal pain within the preceding 8 weeks | 1.09 | (0.82-1.44) | 1.14 | (0.85-1.52) | |
| Spinal pain within the preceding 12 weeks | 1.15 | (0.89-1.47) | 1.26 | (0.97-1.62) | |
| Spinal pain within the preceding 20 weeks | 1.12 | (0.89-1.40) | 1.22 | (0.97-1.54) | |
| ^1^Primary analysis: Is lower extremity pain within the preceding weeks associated with an incident event of spinal pain?  ^2^Reversed analysis: Is spinal pain within the preceding weeks associated with an incident event of lower extremity pain? | | | | | |

| Sensitivity analyses: imputation of missing data using two approaches.  Age and sex adjusted associations, including confidence intervals (CI), between incident events of spinal pain and proportion of weeks with lower extremity (LE) pain the preceding 20 weeks, and the reversed association. | | | |
| --- | --- | --- | --- |
| Missing imputed as  ‘no pain’ | | | Missing imputed as the  same as last week |
| Primary analysis^1^ | | |  |
| Proportion of weeks with LE pain | Odds Ratio  (95% CI) | | Odds Ratio  (95% CI) |
| 0% | 1.00 | | 1.00 |
| 1-50% | 1.35 (1.06-1.72) | | 1.37 (1.08-1.75) |
| 51-100% | 1.59 (1.04-2.43) | | 1.05 (0.62-1.78) |
| Total |  |  |  |
| Reversed analysis^2^ | | |  |
| Proportion of weeks with spinal pain | Odds Ratio  (95% CI) | | Odds Ratio  (95% CI) |
| 0% | 1.00 | | 1.00 |
| 1-50% | 1.15 (0.90-1.55) | | 0.97 (0.81-1.15) |
| 51-100% | 0.94 (0.51-1.72) | | 0.82 (0.49-1.38) |
| Total |  |  |  |

| Age and sex adjusted associations, including confidence intervals (CI), between incident events of spinal pain and proportion of weeks with lower extremity (LE) pain 20 weeks prior to analysis, and the reversed association. | | | | |
| --- | --- | --- | --- | --- |
| Missing imputed as ‘no pain’ | | | Missing imputed as the same as last week | |
| Primary analysis |  |  |  |  |
| Proportion of weeks with LE pain^1^ | n | Hazard ratio  (95% CI) | n | Hazard ratio (95% CI) |
| 0% | 1,008 |  | 987 |  |
| 1-50% | 515 | 1.62 (1.24-2.11) | 543 | 1.71 (1.32-2.23) |
| 51-100% | 122 | 2.07 (1.34-3.20) | 115 | 1.69 (1.07-2.66) |
| Total | 1,645 |  | 1,645 |  |
| Reversed analysis |  |  |  |  |
| Proportion of weeks with spinal pain |  |  |  |  |
| 0% | 1,006 |  | 1,001 |  |
| 1-50% | 168 | 1.42 (1.06-1.92) | 174 | 1.63 (1.19-2.23) |
| 51-100% | 34 | 1.51 (0.82-1.78) | 33 | 0.81 (0.34-1.96) |
| Total | 1,208 |  | 1,208 |  |
| ^1^ Is lower extremity pain within the preceding weeks associated with an incident case of spinal pain?  ^2^ Is spinal pain within the preceding weeks associated with an incident case of lower extremity pain? | | | | |
